# Synergistic Inhibition of Pancreatic Cancer Cell Growth and Migration by Gemcitabine and Withaferin A

**DOI:** 10.3390/biom14091178

**Published:** 2024-09-19

**Authors:** Renata Szydlak

**Affiliations:** Department of Bioinformatics and Telemedicine, Faculty of Medicine, Jagiellonian University Medical College, Medyczna 7, 30-688 Krakow, Poland; renata.szydlak@uj.edu.pl

**Keywords:** pancreatic cancer, gemcitabine, Withaferin A, combined therapy, apoptosis, cell migration, ROS signaling, NF-κB

## Abstract

Pancreatic cancer remains one of the most lethal malignancies due to its aggressive nature and resistance to conventional therapies. This study investigates the anti-proliferative, pro-apoptotic, and anti-migratory effects of Gemcitabine (GC) and Withaferin A (WFA) on pancreatic cancer cell lines PANC-1 and Hs766t. The MTS assay revealed that both compounds effectively inhibit cell proliferation, with WFA showing a stronger effect in Hs766t cells. Flow cytometry analysis demonstrated that GC and WFA, particularly in combination, significantly induce apoptosis in both cell lines. Migration assays confirmed the potent inhibition of cell migration by both compounds, with the combination treatment being the most effective. Furthermore, actin cytoskeleton analysis indicated substantial changes in cell morphology and stiffness, suggesting that GC and WFA disrupt the structural integrity of cancer cells. Additionally, the study highlights a ROS-mediated mechanism underlying the effects of GC and WFA, as evidenced by increased ROS levels following treatment, which were attenuated by N-acetylcysteine. Importantly, NF-κB activity was significantly modulated, with WFA reducing NF-κB activation induced by GC, potentially contributing to the synergistic pro-apoptotic effect of the combination. These findings suggest that the combination of GC and WFA may offer a synergistic therapeutic approach for treating pancreatic cancer by targeting multiple aspects of tumor cell behavior.

## 1. Introduction

Pancreatic cancer is one of the most deadly cancers in the world, with a five-year survival rate of less than 10%, even with recent advances in treatment [1,2]. Its aggressive nature, early metastasis, and resistance to conventional chemotherapy make it a particularly difficult disease to treat [3,4]. Pancreatic ductal adenocarcinoma (PDAC), the most common form of pancreatic cancer, is particularly challenging due to its complex tumor environment, dense stromal tissue, and significant genetic diversity [5]. Current treatments, such as gemcitabine and FOLFIRINOX, offer only modest survival benefits and are often limited by serious side effects and the rapid development of drug resistance [6,7]. As a result, there is growing interest in investigating new treatment combinations that could potentially increase efficacy while reducing toxicity.

Gemcitabine (GC), a nucleoside analog, has long been the cornerstone of chemotherapy for pancreatic cancer [8]. It acts by inhibiting DNA synthesis, leading to apoptosis in rapidly dividing cells [9]. However, the clinical efficacy of gemcitabine is limited by several factors, including the development of drug resistance and its limited ability to induce complete tumor remission [4]. This resistance is often associated with the activation of survival pathways, mutations in tumor suppressor genes, and the intrinsic characteristics of pancreatic cancer cells, such as their highly desmoplastic stroma, which acts as a physical barrier to drug delivery [9,10].

To overcome these limitations, combination therapies involving gemcitabine and other chemotherapeutic agents or molecularly targeted drugs have been explored [9,11,12,13,14]. The rationale behind combination therapy is to exploit the potential synergistic effects between drugs, thereby enhancing their efficacy while potentially reducing the doses required to achieve therapeutic outcomes. However, finding effective drug combinations that significantly improve patient outcomes remains challenging.

Among the novel approaches being explored, the combination of conventional chemotherapeutic drugs with natural compounds has gained considerable interest [15,16]. Natural compounds, which are sourced from plants and other organisms, have been acknowledged for their therapeutic potential, particularly due to their capacity to target multiple signaling pathways and influence cellular processes that are essential for cancer progression [16,17,18]. Withaferin A (WFA), a steroidal lactone obtained from the plant Withania somnifera, is one such compound that has demonstrated potential in preclinical studies against various cancers, including pancreatic cancer [19,20,21]. WFA’s diverse biological activities, such as anti-inflammatory, anti-angiogenic, and pro-apoptotic effects, make it a compelling option for combination therapy [19,22,23,24].

The potential of WFA to enhance the efficacy of conventional chemotherapeutic agents has made it a subject of interest in the development of combination therapies [25,26]. WFA and GC can act synergistically in cancer treatment due to complementary mechanisms that lead to increased DNA damage and induction of apoptosis in cancer cells [9]. The mechanisms by which WFA exerts its anti-cancer effects include the inactivation of Akt and NF-κB [27,28], the generation of ROS [29], cell cycle arrest in the G2/M phase [30], the induction of caspases and other pro-apoptotic proteins [31], and the inhibition of key survival proteins such as HSP90, Notch-1, and HPV oncoproteins E6 and E7 [18]. On the other hand, GC disrupts DNA synthesis, inhibiting the growth of cancer cells and leading to their death [9]. In the context of increased oxidative stress induced by WFA, cancer cells become more sensitive to disruptions in DNA synthesis, which enhances the effect of gemcitabine. The combined use of WFA and GC may result in increased ROS production and further DNA damage. WFA’s action on various signaling pathways and survival proteins can make cancer cells more susceptible to the effects of GC.

The current study aims to further elucidate the synergistic effects of gemcitabine and WFA on pancreatic cancer cells. Previous studies have shown that both WFA and GC can inhibit the proliferation of cancer cells, but the extent of their combined effects on cell viability and proliferation has not been thoroughly investigated [8,9,20].

Moreover, the ability of cancer cells to migrate and invade surrounding tissues is a key determinant of metastasis, the leading cause of cancer-related deaths [32]. Therefore, understanding how WFA and GC influence the migratory capacity of pancreatic cancer cells is critical for assessing their potential to prevent or limit metastasis. Given the central role of the actin cytoskeleton in cell migration, this study also examines the effects of WFA and GC on the actin cytoskeleton, as well as the resulting changes in cell morphology and stiffness.

The mechanical properties of cancer cells, such as stiffness and deformability, have been increasingly recognized as important factors in cancer progression and metastasis [33,34]. Softer cancer cells are generally believed to be more invasive due to their ability to navigate through the dense extracellular matrix and narrow spaces within tissues [33]. However, in the context of pancreatic cancer, this paradigm has been challenged, as stiffer cells have been shown to be more invasive [35]. This study investigates how WFA and GC influence the mechanical properties of pancreatic cancer cells and explores the potential implications for their migratory and invasive behavior. Additionally, the actin cytoskeleton plays a crucial role in regulating not only cell mechanics but also intracellular signaling pathways, including the generation of reactive oxygen species (ROS) [36]. Disruption of the actin cytoskeleton can lead to increased ROS production, which is linked to oxidative stress and the promotion of apoptosis.

Reactive oxygen species (ROS) are crucial signaling molecules that play a role in various cellular functions such as proliferation, differentiation, and apoptosis [37]. Although elevated ROS levels can cause oxidative stress, leading to cellular damage and death, they can also enhance cancer cell survival and resistance to treatment [38]. The dual nature of ROS in cancer highlights the necessity of understanding how therapeutic agents affect ROS levels and related signaling pathways. This study investigates how WFA and GC influence ROS-mediated mechanisms in pancreatic cancer cells, shedding light on their cytotoxic effects and the potential of ROS modulation as a therapeutic approach.

The NF-κB pathway, in particular, plays a crucial role in pancreatic cancer progression, survival, and chemoresistance [39]. NF-κB is a transcription factor that regulates genes involved in inflammation, cell survival, and proliferation, and its constitutive activation is common in pancreatic cancer. Activation of the NF-κB pathway is associated with resistance to apoptosis, increased cell survival, and enhanced tumor aggressiveness [39]. WFA has been shown to inhibit NF-κB activation, thereby promoting apoptosis and sensitizing cancer cells to chemotherapeutic agents [40]. By combining WFA with gemcitabine, it is possible to not only enhance the direct cytotoxic effects of chemotherapy but also suppress key survival pathways mediated by NF-κB. This dual approach could be particularly effective in overcoming drug resistance and improving treatment outcomes in pancreatic cancer patients.

This study aims to clarify the combined impact of WFA and GC on pancreatic cancer cells, particularly in relation to cell proliferation, apoptosis, migration, cytoskeletal dynamics, and ROS-mediated signaling. By exploring these factors, this research intends to offer a thorough understanding of the therapeutic potential of WFA and GC in treating pancreatic cancer, setting the stage for future preclinical and clinical investigations that could pave the way for more effective combination therapies for this challenging disease.

## 2. Materials and Methods

### 2.1. Cell Lines

Human pancreatic cancer cell lines PANC-1 (primary cancer) and Hs766t (metastasis) were purchased in ATCC (American Type Culture Collection, Manassas, VA, USA). Cells were cultivated in high-glucose DMEM medium (Lonza Group Ltd., Basel, Switzerland) supplemented with 10% fetal bovine serum (FBS, Sigma, St. Louis, MO, USA) and 50 µg/mL gentamicin (Lonza Group Ltd., Basel, Switzerland) at 37 °C, 5% CO^2^ and 95% humidity. Cells were passaged two or three times a week. Cell lines were routinely observed under an optical microscope (Olympus, Tokyo, Japan).

### 2.2. Treatments of the Cells

Stock solutions of Whitaferin A (WFA) (Sigma, St. Louis, MO, USA) were prepared by dissolving the powder in dimethyl sulfoxide (DMSO) (Sigma, St. Louis, MO, USA) to a final concentration of 10 mM. Stock solution of gemcitabine (Selleck Chemicals, Houston, TX, USA) was prepared by dissolving compounds in DMSO to a final concentration of 1 mM. The final solutions were stored at −20 °C. DMEM was used to dilute the stock solutions to obtain working concentrations (WFA: 0.01–100 µM; GC: 0.01–100 nM). As a negative control, the administered cells were cultured under analogous conditions in DMEM.

### 2.3. Assessment of Chemosensitivity of Cells

All cells were seeded into 96-well plates at concentrations of 8 × 10^3^ cells/well and 100 µL of 10% FBS (Sigma, St. Louis, MO, USA) DMEM/well. After a 24-h incubation, the medium was replaced by DMEM with 1% FBS and containing WFA in the concentration range of 0.01–100 µM or GC in the concentration range of 0.01–100 nM. The incubation was continued for another 48 h at 37 °C in a humid atmosphere. An MTS assay (Promega, Madison, WI, USA) was used to determine the effect of WFA and GC on pancreatic cancer cell viability. Absorbance was measured using a microplate reader (BioTek Instruments Inc, Winooski, VT, USA). Six replicate wells were used for each experiment. The effects of WFA and GC were expressed as relative (vs. control) decrease in cell growth determined after 48 h of incubation with the test compound.

### 2.4. Apoptosis Analysis by Flow Cytometry

Pancreatic cancer cells were seeded and cultured in a six-well plate with test drugs. After a 48-h incubation with drugs, according to the manufacturer’s protocol, apoptosis was analyzed using the Annexin V PE Apoptosis Detection Kit (BD Biosciences, Franklin Lakes, NJ, USA). Briefly, treated tumor cells were harvested in 6-well plates, washed twice with ice-cold phosphate-buffered saline (PBS, Sigma-Aldrich, St. Louis, MI, USA), and centrifuged at 1000× *g* for 5 min, then 1 × 10^5^ cells were resuspended in 100 µL of binding buffer containing 5 µL of annexin V-FITC and 5 µL of propidium iodine (PI) in the dark. After 15 min, 400 µL of buffer was added before cytometric analysis. Quantification of apoptotic cells was analyzed by flow cytometry on a BD FACS Canto^TM^ (Becton Dickinson, New York, NY, USA).

### 2.5. Chemotaxis Assay

Chemotaxis of pancreatic cancer cells to 10% FBS in DMEM was evaluated using a modified Boyden chamber with 8 µm pore polycarbonate membrane inserts (Transwell; Corning Life Sciences, PZ HTL SA, Warsaw, Poland) and chemotactic cells were visualized using calcein-AM staining (BD Biosciences, Franklin Lakes, NJ, USA). BSA DMEM 0.5% medium was used as a negative control. Prior to the experiment, pancreatic cancer cells were treated with WFA or GC or the composition of GC and WFA at an IC50 concentration for 48 h.

### 2.6. Fluorescence Microscopy

For fluorescent staining, cells were seeded onto 15 × 15 mm coverslips (FISHERfinets^TM^, Drive Pittsburgh, PA, USA) placed in the wells of 12-well plates and cultured for at least 24 h. Then the culture medium was replaced with the fresh one containing tested drugs and cultivated for 48 h at 37 °C and 95% humidity. The cells were then washed twice with PBS containing Ca^2+^ and Mg^2+^, and fixed in 3.7% formaldehyde for 20 min at 37 °C. Following fixation, the cells were permeabilized with a 0.1% Triton X-100 solution in PBS with Ca^2+^ and Mg^2+^ for 5 min at 37 °C, and then blocked with a 3% BSA solution in PBS for 1 h at 37 °C. Between each of these steps, the cells were washed twice with PBS containing Ca^2+^ and Mg^2+^. For cytoskeletal architecture analyses, actin microfilaments were stained using Alexa-Fluor 488 conjugated with phalloidin (Invitrogen, Waltham, MA, USA). Hoechst 34,580 at a concentration of 1 μg/mL (No. B2883, Sigma-Aldrich, St. Louis, MI, USA) was used to visualize cell nuclei.

All fluorescence images were recorded using an Olympus IX70 microscope (Olympus, Tokyo, Japan) and a Canon EOS1100 digital photo camera.

### 2.7. F-Actin Content Determination

To assess F-actin content, cells were initially seeded at a density of 5000 cells per well in black-walled 24-well plates. After 24 h, the culture medium was renewed with one containing the test drugs, and the cells were cultured for 48 h at 37 °C with 95% humidity. The cells were then stained with Alexa Fluor 488-conjugated phalloidin (0.033 μM in PBS, Molecular Probes, Eugene, OR, USA). The binding of phalloidin to F-actin was then quantified using a SpectraMax Gemini microplate reader (Molecular Devices, Sunnyvale, CA, USA) with the appropriate excitation and emission settings for Alexa Fluor 488 (Ex 488 nm/Em 518 nm).

### 2.8. AFM Measurements

For AFM measurements, cells were seeded onto 15 × 15 mm coverslips (FISHERfinets^TM^, Drive Pittsburgh, PA, USA) placed in the wells of 12-well plates with 2 mL of DMEM supplemented with 10% FBS, and cultured for at least 24 h. The medium was then replaced with fresh DMEM containing 1% FBS. To evaluate the effect of specific compounds or their combinations on mechanical properties, GC, WFA, or a combination of GC and WFA at IC50 doses were added to the culture medium. A sample containing only DMEM with 1% FBS served as a control (NTC). AFM measurements were performed 48 h after the addition of the compounds, with the culture medium changed daily to maintain a constant concentration of the compounds.

Measurements were conducted using a FlexAFM microscope (Nanosurf, Liestal, Switzerland) at 37 °C. OTR-4–10 cantilevers (Bruker, Billerica, MA, USA) with a nominal elastic constant of 0.02 N/m were used, and the elastic constant of the cantilever was calibrated before measurements. For each sample (experimental conditions, cell type), 20 cells were measured in each of 3 independent replicates (totaling 60 cells). For each cell, 25 force–distance curves were obtained over an area of 5 × 5 µm^2^. The loading force was set at 1.5 nN, and the loading rate was 8 µm/s. Each sample measurement took approximately 60 min.

The data collected during the AFM measurements were extracted using the Nanosurf 1.5.0 Python package. Subsequently, MATLAB R2022B was used for analysis.

### 2.9. ROS Analysis

To assess intracellular reactive oxygen species (ROS) levels, the fluorescent probe 2′,7′-dichlorofluorescin diacetate (DCFH-DA) was used. This technique tracks ROS by measuring the fluorescence emitted when DCFH-DA is oxidized. Pancreatic cancer cells were first seeded into a 12-well plate and allowed to adhere overnight. The next day, the cells were washed in PBS to remove traces of the original medium, treated with 10 μM DCFH-DA (purchased from Sigma-Aldrich, St. Louis, MO, USA) in a serum-free medium for 30 min at 37 °C in the dark. Within the cells, DCFH-DA is deacetylated by cellular esterases into 2′,7′-dichlorofluorescin (DCFH), which is non-fluorescent. Post-incubation, the cells were rinsed three times with PBS to remove any remaining DCFH-DA outside the cells, leaving only intracellular DCFH available for oxidation by ROS, forming the fluorescent compound 2′,7′-dichlorofluorescin (DCF). The cells were then subjected to various treatments, including a control (no treatment), IC50 doses of GC and WFA individually, a combination of GC and WFA, and a combination of GC, WFA, and 1 mM NAC. After 48 h of treatment, the cells were analyzed using a flow cytometry (Becton Dickinson, New York, NY, USA).

### 2.10. Cell Transfection with NF-κB Luciferase Reporter Construct

Cells were transfected with the pGL4.32 [luc2P/NF-κB-RE/Hygro] luciferase reporter plasmid (Promega, Madison, WI, USA) and the Renilla luciferase control plasmid pRL-TK (Promega, Madison, WI, USA) using Lipofectamine 3000 (Thermo Fisher Scientific, Drive Pittsburgh, PA, USA) according to the manufacturer’s protocol. Cells were seeded at 2 × 10^5^ cells per well in 6-well plates 24 h prior to transfection. For each well, 2.5 µg of pGL4.32 and 0.25 µg of pRL-TK were mixed with 5 µL P3000 reagent in 125 µL Opti-MEM (Gibco, Thermo Fisher Scientific, Drive Pittsburgh, PA, USA). The mixture was combined with 5 µL Lipofectamine 3000 and incubated for 10 min before being added to the cells. After 6 h, the medium was replaced with a completely fresh medium.

A total of 24 h post-transfection, cells were treated with Gemcitabine (Sigma-Aldrich, St. Louis, MI, USA) and Withaferin A (Sigma-Aldrich, St. Louis, MI, USA) dissolved in DMSO (0.1% final DMSO concentration). Following 24 h of treatment, cells were lysed using Passive Lysis Buffer (Promega, Madison, WI, USA), and luciferase activity was measured with the Dual-Luciferase Reporter Assay System (Promega, Madison, WI, USA) using a GloMax^®^ 96 Microplate Luminometer (Promega, Madison, WI, USA). Firefly luciferase activity was normalized to Renilla luciferase activity. Data were expressed as the ratio of normalized luciferase activity in treated samples to control samples (0.1% DMSO), and presented as mean ± SD. 

### 2.11. Statistical Analysis

Statistical analysis was conducted by comparing means with standard errors, and the Student’s *t*-test was applied. A *p* value of ≤ 0.05 was deemed statistically significant. Each experimental group was replicated at least three times.

## 3. Results

### 3.1. GC and WFA Show Anti-Proliferative Effects on Pancreatic Cancer Cells

**Figure 1:**
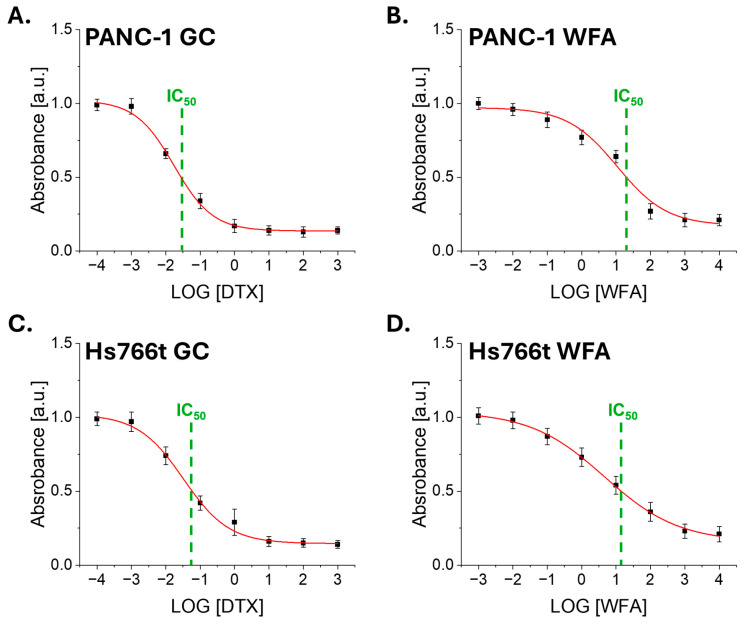
The proliferation of pancreatic cancer cells was analyzed after treatment with different concentrations of Gemcitabine and Withaferin A. (**A**,**B**) Dose–response curves for PANC-1 cells treated with Gemcitabine (**A**) and Withaferin A (**B**). (**C**,**D**) Dose–response curves for Hs766t cells treated with Gemcitabine (**C**) and Withaferin A (**D**). The IC50 values are marked by a green line. Untreated pancreatic cancer cells served as the control group. Data are presented as mean ± standard deviation (s.d.) from at least *n* = 3 measurements.

Proliferative activity is one of the characteristics of tumor cells, crucial for tumor promotion. In order to determine to what extent the synergistic cytostatic effects of GC and WFA translate into long-term effects of both drugs on cell proliferation, changes in cell growth were analyzed after a 48-h incubation with these compounds (Figure 1). In order to assess the impact of Withaferin A (WFA) and Gemcibatine (GC) on the proliferative activity of PANC-1 and Hs766t cancer cell lines, an MTS assay was conducted using concentrations ranging from 0.01 to 100 µM for WFA, and an equivalent for GC. These analyses confirmed the high sensitivity of PANC-1 and Hs766t cells to gemcitabine and Withaferin A (Table 1).

### 3.2. GC and WFA Induce Apoptosis in Pancreatic Cancer Cells

**Figure 2:**
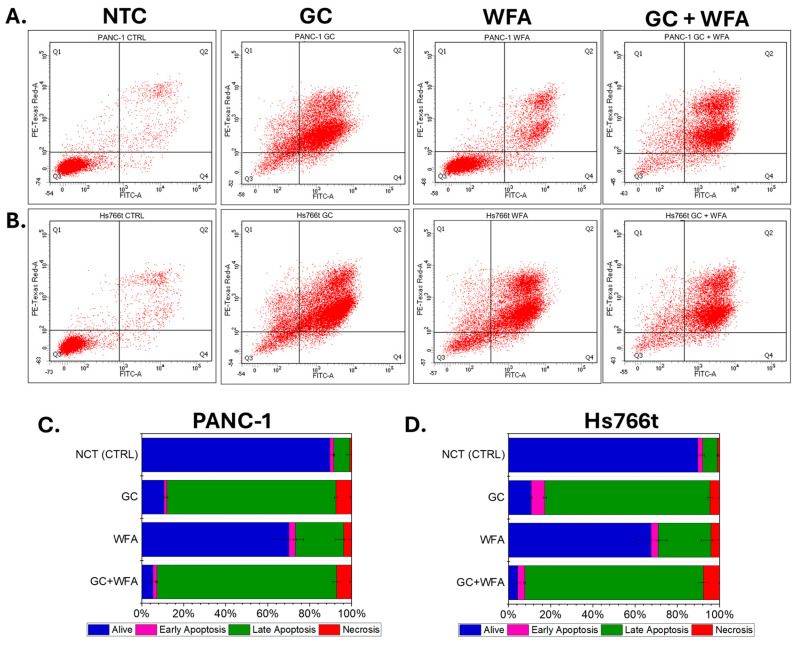
The effect of GC, WFA, and GC + WFA on apoptosis and necrosis in PANC-1 and Hs766t cells. Flow cytometry using Annexin-V and PI staining is depicted in a dot-plot graph, which shows four distinct cell states: alive cells—Q3, necrotic cells—Q1, early apoptotic cells—Q4, and late apoptotic cells—Q2. Pancreatic cancer cells PANC-1 (**A**) and Hs766t (**B**) were exposed to GC, WFA, and a combination of GC and WFA at IC50 concentrations. The cumulative bar charts illustrate the distribution of PANC-1 (**C**) and Hs766t (**D**) cell states after 48 h of treatment. The statistical significance between the samples was assessed using the Student’s *t*-test.

In the next step of the study, the effect of GC and WFA, as well as the combination of GC and WFA (all at IC50 concentrations), was investigated on the survival of pancreatic cancer cells PANC-1 and Hs766t lines. Annexin V and propidium iodide staining, followed by FACS analysis, were used to precisely determine the levels of apoptosis and necrosis in the cells (Figure 2).

The FACS analysis results show the effects of gemcibatine (GC) and Withaferin A (WFA) on two pancreatic cancer cell lines, PANC-1 and Hs766t. For the PANC-1 cell line, the control group (NCT) showed that 89.6% of the cells were alive, 1.8% were in early apoptosis, 7.67% were in late apoptosis, and 0.93% were necrotic. Treatment with GC + WFA resulted in a significant reduction in cell viability, with only 5.43% of the cells remaining alive, 1.83% in early apoptosis, 85.57% in late apoptosis, and 7.17% necrotic. In comparison, WFA alone resulted in 70.13% of the cells being alive, 3% in early apoptosis, 23% in late apoptosis, and 3.87% necrotic. Treatment with GC alone led to 10.5% of the cells being alive, 1.43% in early apoptosis, 80.57% in late apoptosis, and 7.5% necrotic.

For the Hs766t cell line, the control group (NCT) showed that 89.67% of the cells were alive, 2.23% were in early apoptosis, 7.1% were in late apoptosis, and 1% were necrotic. When treated with GC + WFA, the cell viability dropped significantly, with only 4.33% of the cells remaining alive, 3.33% in early apoptosis, 84.63% in late apoptosis, and 7.71% necrotic. WFA treatment alone resulted in 67.53% of the cells being alive, 3.3% in early apoptosis, 25.04% in late apoptosis, and 4.13% necrotic. GC treatment alone led to 10.8% of the cells being alive, 6.4% in early apoptosis, 78.23% in late apoptosis, and 4.57% necrotic.

The FACS analysis demonstrated that the combination of GC and WFA led to the most significant increase in the number of cells in late apoptosis and necrosis in both cell lines, suggesting a synergistic effect of these compounds in inducing cancer cell death. These conclusions are supported by the significantly reduced number of living cells and the increased number of apoptotic and necrotic cells in the groups treated with GC + WFA compared to the control groups and groups treated with the individual compounds. These results suggest that the combination of GC and WFA is a potent inducer of apoptosis in pancreatic cancer cells.

### 3.3. GC and WFA Inhibit Migration of Pancreatic Cancer Cells

The ability of cancer cells for active migration is one of the main determinants of the invasiveness of cancer cells, and can be used as a parameter for predicting the effects of potential chemotherapy drugs on the well-being of cancer cells. To determine the effect of gemcitabine (GC) and Withaferin A (WFA) on pancreatic cancer cells, the migration activity of PANC-1 and Hs766t cells was analyzed after separate or simultaneous administration of both compounds. For this purpose, the Boyden chamber migration assay was used.

**Figure 3:**
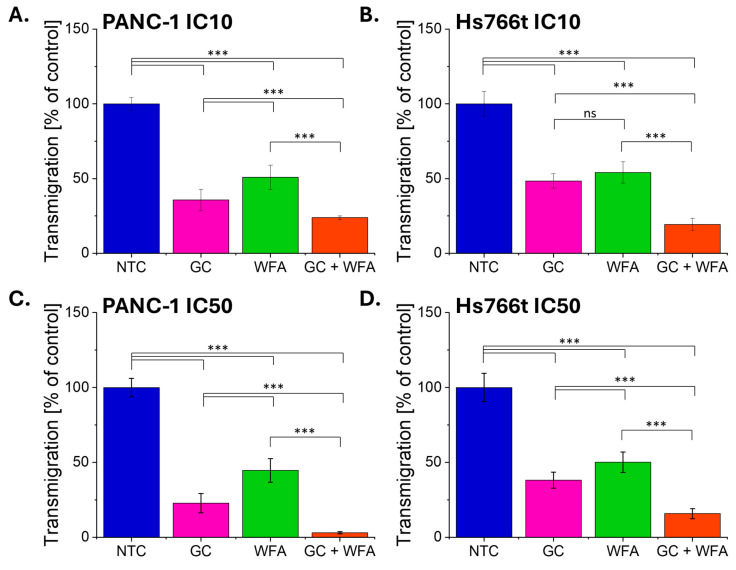
The effect of GC, WFA, and GC + WFA on the migration potential of PANC-1 and Hs766t cells. The efficiency of transmigration through 8 µm pores by PANC-1 (**A**,**C**) and Hs766t (**B**,**D**) cells at IC10 (**A**,**B**) and IC50 (**C**,**D**) is presented as a percentage of the control (non-treated cells) from three independent experiments. Statistical significance between non-treated and treated samples was evaluated using an unpaired Student’s *t*-test: ns—non-significant (*p* > 0.05); *** *p* < 0.001.

The results showed that gemcitabine significantly reduced the migratory activity of both PANC-1 and Hs766t pancreatic cancer cells (Figure 3). Withaferin A also impaired the migration of these cells (Figure 3). However, the strongest inhibition was observed when both compounds were co-administered (Figure 3).

### 3.4. The Impact of GC and WFA on Actin Cytoskeleton Mechanical Properties of Pancreatic Cancer Cells

**Figure 4:**
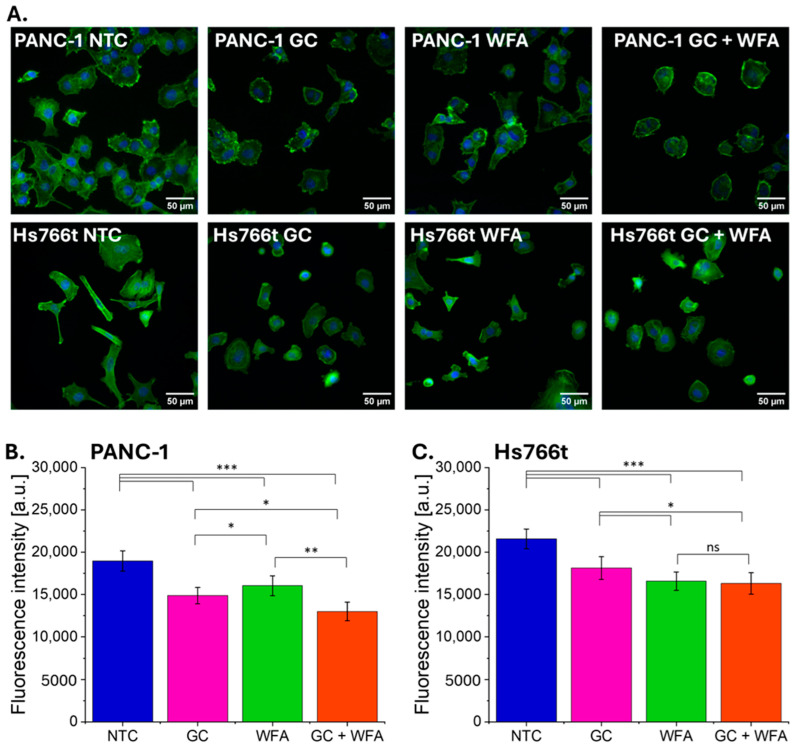
Response of the pancreatic cancer cell cytoskeleton to the drug treatment (**A**). PANC-1 and Hs766t cells after 48 h of treatment with GC, WFA, and GC + WFA, as well as NTC (control). Actin filaments (F-actin, Alexa Fluor 488) and cell nuclei (Hoechst 34580) (A). Scalebar = 50 µm. The change in phalloidin binding, corresponding to F-actin content of PANC-1 (**B**) and Hs766t (**C**), after treatment with GC, WFA, and the combination of GC + WFA, presented as mean ± standard deviation. Statistical verification of the results was performed using Student’s *t*-test: ns—non-significant (*p* > 0.05); * 0.01 < *p* < 0.05, ** 0.001 < *p* < 0.01, *** *p* < 0.001.

Given the cytoskeleton’s crucial role in cell migration, the next step was to evaluate the impact of the tested compounds on the structure of the actin cytoskeleton. Figure 4 displays fluorescence images of PANC-1 and Hs766t cells: untreated (NCT), treated with GC, treated with WFA, and combinations of these compounds at their IC50 doses.

The F-actin content was also assessed by measuring the intensity of phalloidin binding, which reflects F-actin content in two cell lines, PANC-1 and Hs766t, following treatment with GC, WFA, and their combination.

There were significant changes in cell shape and actin cytoskeleton structure after exposure to both compounds. In particular, the cells changed from a spindle shape to an oval morphology. Interestingly, the effect on cell morphology and actin cytoskeleton was less significant when gemcitabine and Withaferin A were administered separately. In PANC-1 cells, the combination treatment had a more pronounced effect compared to either compound alone. However, in Hs766t cells, no significant differences were observed between cells treated with WFA alone and those treated with the combination of GC and WFA. Fragmentation of actin filaments was also observed. These results suggest that Withaferin A enhances the anti-migratory effect of gemcitabine on pancreatic cancer cells, indicating a possible synergistic interaction between the two compounds leading to F-actin depolymerization (Figure 3 and Figure 4).

A crucial role in cellular migratory capabilities and invasive potential plays deformability of cancer cells. The mechanotype of cells, which encompasses their mechanical properties, can influence how cancer cells navigate through dense tissues and narrow blood vessels during metastasis. While many studies have shown that more deformable (softer) cancer cells tend to have greater invasive potential, in the case of pancreatic ductal adenocarcinoma (PDAC), previous findings suggest that stiffer cells may exhibit higher invasiveness [35]. Cell stiffness can affect their ability to active changes of their shape during movement in mechanically confined environments and interact with the extracellular matrix, facilitating migration and invasion.

**Figure 5:**
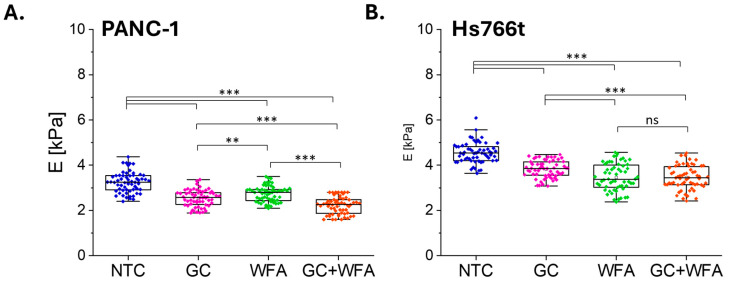
The effect of GC, WFA, and GC + WFA on stiffness of pancreatic cancer cells. Boxplots showing the Young’s modulus values of pancreatic cancer cells PANC-1 (**A**) and Hs766t (**B**) treated with GC, WFA, and GC + WFA, as well as the NTC (control). Each point represents the mean Young’s modulus value calculated from data collected from a single cell. The box plots display the median (line), mean (open square), and standard deviation (box). Statistical analysis was performed using the Student’s *t*-test. Statistical significance was determined at a significance level of 0.05 (ns—no significant difference, ** *p* < 0.01, *** *p* < 0.001).

In this step, also the impact of gemcitabine (GC) and Withaferin (WFA), as well as the combination of these compounds, on the deformability of pancreatic cancer cell lines PANC-1 and Hs766t was investigated (Figure 5).

AFM measurements revealed that the stiffness of PANC-1 cells decreased after treatment with gemcitabine and Withaferin A, both individually and in combination. Specifically, the stiffness of untreated PANC-1 cells (NTC) was measured at 3.239 kPa. Treatment with gemcitabine reduced the stiffness to 2.541 kPa, while Withaferin treatment resulted in a stiffness of 2.737 kPa. The combination of gemcitabine and Withaferin further decreased the stiffness to 2.217 kPa.

Similarly, in Hs766t cells, a reduction in stiffness was observed following treatment. Untreated Hs766t cells exhibited a stiffness of 4.545 kPa. Treatment with gemcitabine lowered the stiffness to 3.819 kPa, and Withaferin treatment reduced it to 3.455 kPa. The combination treatment resulted in a stiffness of 3.458 kPa.

These findings demonstrate that treatment with gemcitabine and Withaferin reduces the stiffness of pancreatic cancer cells in both examined cell lines (Figure 5). The combination of these compounds results in a greater reduction in cell stiffness compared to individual treatments, suggesting a potential synergistic effect in altering the mechanotype of the cells. Further studies are required to elucidate how these changes in cell stiffness influence their migratory and invasive capabilities, and to understand the underlying molecular mechanisms involved.

### 3.5. ROS-Mediated Mechanism of Action of Gemcibatine and Withaferin A

**Figure 6:**
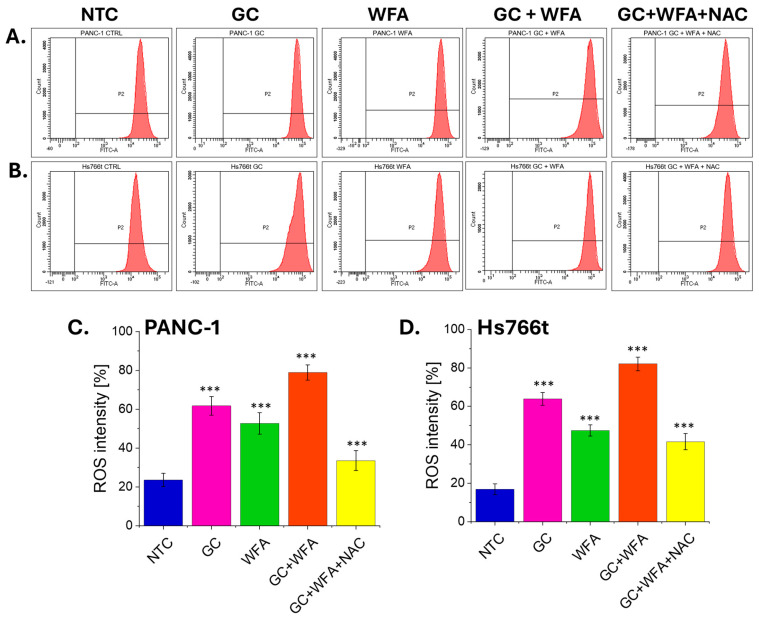
The effect of GC, WFA, GC + WFA, and GC + WFA + NAC on ROS production pancreatic cancer cells. Flow cytometry analysis of ROS generation in PANC-1 cells (**A**,**C**), and Hs766t cells (**B**,**D**) (representative histogram from three independent experiments). Data are presented as mean ± st. dev. from three independent experiments. Statistical analysis was performed using the Student’s *t*-test. Statistical significance was determined at a significance level of 0.05 (ns—no significant difference, *** *p* < 0.001).

In order to understand the mechanism of action of gemcitabine (GC) and Withaferin A (WFA) on pancreatic cancer cells, the analysis of ROS was performed (Figure 6). Investigating ROS levels was crucial because ROS are key signaling molecules that regulate various cellular processes, including proliferation, differentiation, and apoptosis, and their dysregulation contributes to tumor progression and therapy resistance.

The ROS intensity measurements using flow cytometry in PANC-1 and Hs766t pancreatic cancer cell lines show notable differences across treatments, indicating that ROS plays a crucial role in the signaling pathways activated by the compounds. In PANC-1 cells, the control (NTC) has a baseline ROS intensity of 23.55. Treatment with gemcitabine (GC) significantly increases ROS intensity to 61.74, while Withaferin A (WFA) elevates it to 52.73. The combination of GC and WFA further amplifies ROS intensity to 78.91, suggesting a synergistic effect in ROS production. When N-acetylcysteine (NAC), an antioxidant, is added to the combination treatment, the ROS intensity is reduced to 33.53. This reduction indicates that NAC mitigates ROS production, supporting the idea that the observed effects of GC and WFA are mediated through ROS signaling.

Similarly, in Hs766t cells, the control has a baseline ROS intensity of 16.92. GC treatment raises ROS intensity substantially to 63.85, and WFA treatment increases it to 47.46. The combination of GC and WFA leads to the highest ROS intensity of 82.08. The addition of NAC to the combination treatment decreases ROS intensity to 41.62, demonstrating that the antioxidant effect of NAC reduces the ROS levels induced by GC and WFA. These results confirm that the signaling pathways activated by GC and WFA in pancreatic cancer cells are mediated through ROS, as evidenced by the attenuation of ROS levels upon NAC treatment.

### 3.6. GC and WFA Modulates the NF-κB Activity in Pancreatic Cancer Cells

**Figure 7:**
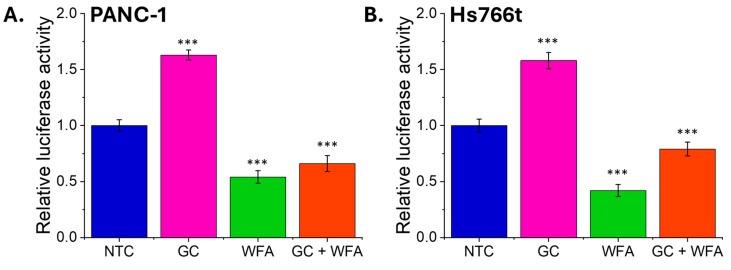
The effect of GC, WFA, and GC + WFA on NF-κB activity in pancreatic cancer cells. Relative luciferase activity in PANC-1 cells (**A**) and Hs766t cells (**B**) (representative histogram from three independent experiments). Data are presented as mean ± st. dev. from three independent experiments. Statistical analysis was performed using the Student’s *t*-test. Statistical significance was determined at a significance level of 0.05 (ns—no significant difference, *** *p* < 0.001).

The nuclear factor kappa-light-chain-enhancer of the activated B cell (NF-κB) pathway plays a pivotal role in the survival, proliferation, and resistance to apoptosis of cancer cells. To evaluate the influence of GC and WFA on NF-κB activity, a luciferase reporter assay was performed using the PANC-1 and Hs766t pancreatic cancer cell lines transfected with an NF-κB luciferase reporter construct. The findings demonstrated that, in both cell lines, the relative luciferase activity, which reflects NF-κB activity, was markedly elevated following treatment with GC (Figure 7). In contrast, treatment with WFA resulted in a notable decline in luciferase activity, particularly when combined with GC.

Treatment with GC alone resulted in a significant increase in NF-κB activity to 1.63 (±0.045), indicating that GC strongly activates NF-κB, which aligns with its known potential to promote cell survival and contribute to chemoresistance. In contrast, WFA alone reduced NF-κB activity to 0.54 (±0.056), demonstrating its inhibitory effect on this pathway. When both GC and WFA were combined, the NF-κB activity decreased to 0.66 (±0.071), suggesting that WFA partially counteracts the GC-induced activation of NF-κB, which may lead to enhanced apoptosis in these cells.

In the Hs766t cell line, the pattern was similar. The GC treatment alone increased NF-κB activity to 1.58 (±0.073), again underscoring GC’s role in NF-κB activation. WFA alone significantly reduced NF-κB activity to 0.42 (±0.054), highlighting its potential to suppress NF-κB-mediated survival signals. The combination of GC and WFA resulted in an NF-κB activity of 0.79 (±0.062), further supporting the hypothesis that WFA can mitigate the NF-κB activation induced by GC.

These findings suggest that the apoptotic synergy observed when GC and WFA are used together could be attributed to WFA’s ability to inhibit NF-κB activity, thereby counteracting the pro-survival effects induced by GC. This modulation of NF-κB by WFA could be critical in overcoming chemoresistance, making the GC and WFA combination a promising therapeutic strategy in treating pancreatic cancer. The ability of WFA to lower NF-κB activity, especially in the context of GC treatment, highlights its potential role in enhancing the efficacy of chemotherapy by promoting apoptosis through the suppression of NF-κB-mediated survival pathways.

## 4. Discussion

This study explored the synergistic effects of the widely used chemotherapeutic agent gemcitabine and the adaptogen Withaferin A on the biological properties of two pancreatic cancer cell lines: PANC-1, derived from primary tumors, and Hs766t, derived from a metastatic lymph node site. These cell lines represent different stages of pancreatic cancer progression. Notably, Hs766t cells exhibited prominent stress fibers, increased proliferative and migratory activity, and a significantly enhanced ability to transmigrate across mechanical barriers compared to PANC-1 cells. These characteristics align with previous studies, which associate enhanced migratory properties with metastasis formation in cancer cells [41,42]. Furthermore, NTC fluorescence imaging (Figure 4) revealed that Hs766T cells exhibit a more mesenchymal phenotype than PANC-1 cells. Such cellular features, indicative of increased invasive potential, have been observed not only in pancreatic cancer [43], but also in various other cancer types [44].

Previous research has highlighted the efficacy of GC as a standard chemotherapeutic agent in pancreatic cancer due to its ability to incorporate into DNA and inhibit replication, leading to cell death [45,46,47]. WFA, a steroidal lactone derived from Withania somnifera, has also been shown to exhibit anti-proliferative properties in various cancer models, including breast [48], cervical [21], and ovarian cancer [49]. The differential sensitivity between the two cell lines highlights the importance of tailoring treatment strategies to the specific characteristics of different pancreatic cancer cells. Pancreatic cancer is notoriously heterogeneous, with variations in genetic mutations, cellular behaviors, and treatment responses [50,51]. The anti-proliferative results are promising for the development of combination therapies. Gemcitabine is a standard chemotherapy agent for pancreatic cancer, but resistance often develops, limiting its long-term effectiveness [52]. The addition of WFA, which operates through different cellular pathways, could help overcome this resistance, providing a more robust and sustained therapeutic effect. The study suggests that the combination of these agents could be more effective than either alone.

The induction of apoptosis is a critical mechanism by which chemotherapeutic agents exert their anti-cancer effects. This study demonstrated that the combination of GC and WFA significantly increased the proportion of cells undergoing late apoptosis and necrosis in both PANC-1 and Hs766t cell lines. This finding is consistent with previous studies that have shown WFA to enhance apoptosis in cancer cells through multiple pathways, including the activation of caspases, inhibition of NF-κB, and disruption of heat shock protein 90 (HSP90) function [53]. Gemcitabine is known to induce apoptosis by causing DNA damage and activating the p53 pathway [54]. The observed synergistic effect of GC and WFA in promoting apoptosis could be due to their complementary mechanisms of action, which together overwhelm the cancer cells’ survival pathways. These results are particularly relevant for overcoming chemoresistance, a significant challenge in treating pancreatic cancer. Many cancer cells develop mechanisms to evade apoptosis, contributing to resistance to treatments like gemcitabine [55,56]. The addition of WFA, with its ability to activate alternative apoptotic pathways, could provide a way to bypass or overcome these resistance mechanisms.

On the other hand, a key determinant of metastatic potential is cancer cell migratory activity [57,58]. The obtained results indicate that both GC and WFA significantly impair the migration of PANC-1 and Hs766t cells, with the most pronounced effect observed when both compounds were administered together. The observed effects on the actin cytoskeleton provide a possible explanation for the reduced migratory capacity. The actin cytoskeleton is essential for maintaining cell shape and enabling movement [59]. The disruption of actin filaments, as observed in this study, likely impairs the cells’ ability to migrate. Interestingly, the combination of GC and WFA led to more pronounced changes in the cytoskeleton in PANC-1 cells than either agent alone, indicating a synergistic effect that could further enhance the anti-migratory properties of the treatment in this cell line. However, this effect was not observed in Hs766t cells, where the combination did not significantly differ from the effects of WFA alone. WFA has previously been shown to inhibit cell migration by modulating the cytoskeleton and decreasing the expression of matrix metalloproteinases (MMPs), which are crucial for extracellular matrix degradation and cancer cell invasion [60]. Similarly, gemcitabine has been reported to reduce the expression of MMPs and inhibit the epithelial-mesenchymal transition (EMT), a process that enhances the migratory capacity of cancer cells [60,61]. The synergistic reduction in cell migration observed with the combination of GC and WFA may result from their combined effects on these pathways, leading to a more significant disruption of the cellular mechanisms that facilitate metastasis.

Also, cancer cell stiffness has been linked to their ability to invade and metastasize [33,62]. Softer, more deformable cells are generally better able to navigate through dense tissues and narrow blood vessels, facilitating metastasis [33]. However, in pancreatic cancer, stiffer cells have also been associated with increased invasiveness, making the relationship between stiffness and cancer behavior more complex [35]. The reduction in cell stiffness observed after treatment with GC and WFA, both individually and in combination, is a noteworthy finding, given the role of cellular mechanotype in cancer progression. The observed depolymerization of F-actin and the change in cell morphology from a spindle shape to an oval shape could impair the cells’ ability to migrate and invade surrounding tissues, thus reducing their metastatic potential. Previous studies have shown that WFA can directly bind to vimentin and other cytoskeletal proteins, leading to cytoskeletal disruption and inhibition of cell motility [63,64]. Gemcitabine’s impact on the cytoskeleton has been less studied, but its ability to reduce cell stiffness, as shown in this study (Figure 5), suggests that it may also affect cytoskeletal dynamics. The synergy between the two agents in reducing stiffness could be particularly important, as it suggests that the combination treatment may be more effective at altering the mechanotype than either agent alone.

The role of reactive oxygen species (ROS) in the mechanism of action of GC and WFA was also explored in this study. ROS are key signaling molecules involved in various cellular processes, including apoptosis [65]. Excessive ROS can lead to oxidative stress, damaging cellular components and ultimately leading to cell death. This study reveals that the combination of GC and WFA significantly increases ROS production in pancreatic cancer cells. This increase in ROS suggests that oxidative stress may be a significant factor in the anti-proliferative and pro-apoptotic effects of WFA and GC. WFA has been reported to induce oxidative stress by disrupting mitochondrial function and inhibiting antioxidant defenses, leading to increased ROS levels and apoptosis [53,66]. Similarly, gemcitabine has been shown to increase ROS production, further enhancing its cytotoxic effects [67,68]. The reduction in ROS levels observed with the addition of the antioxidant N-acetylcysteine (NAC) further supports the role of ROS in the mechanism of action of these compounds. By mitigating the increase in ROS, NAC reduced the cytotoxic effects of GC and WFA, suggesting that the combination of these agents exerts its effects, at least in part, through ROS-mediated pathways. By exploiting the increased ROS levels induced by GC and WFA, it may be possible to enhance their cytotoxic effects, particularly in cancer cells that are resistant to other forms of treatment. Additionally, the combination of ROS-modulating agents with GC and WFA could be explored as a potential strategy to maximize therapeutic efficacy while minimizing side effects.

The modulation of NF-κB activity by GC and WFA presents a compelling explanation for the enhanced apoptosis observed with the combination treatment. The reduction in NF-κB activity by WFA, particularly when combined with GC, suggests that WFA may counteract GC-induced NF-κB activation, which is typically associated with cell survival and chemoresistance. This finding highlights the potential of WFA to enhance the pro-apoptotic effects of GC by inhibiting NF-κB-mediated survival pathways. Given the central role of NF-κB in cancer progression and therapy resistance, the ability of WFA to suppress NF-κB activity could represent a critical mechanism by which it potentiates the efficacy of GC in treating pancreatic cancer.

Interestingly, there is a well-documented relationship between ROS and NF-κB signaling [69]. However, in some cases, excessive ROS can lead to the inhibition of NF-κB activity, particularly when oxidative stress overwhelms the cell’s antioxidant defenses, resulting in apoptosis [70]. In this study, WFA’s ability to reduce NF-κB activity, even in the presence of elevated ROS levels induced by GC, suggests that WFA may disrupt the ROS-mediated activation of NF-κB, thereby tipping the balance towards apoptosis rather than survival. This interaction between ROS and NF-κB could be a crucial mechanism by which WFA enhances the pro-apoptotic effects of GC, making the combination treatment more effective in inducing cancer cell death.

The demonstrated synergy between GC and WFA in inhibiting proliferation, inducing apoptosis, reducing migration, and altering cell stiffness suggests that this combination could be a powerful therapeutic approach. The ability to target multiple aspects of cancer cell biology simultaneously is particularly important in the context of pancreatic cancer, which is known for its complexity and resistance to treatment.

However, several questions remain that warrant further investigation. First, the molecular mechanisms underlying the synergistic effects of GC and WFA need to be elucidated. Understanding these mechanisms could provide valuable insights into how to optimize the combination therapy and identify other compounds that could enhance its efficacy.

Second, while the study’s in vitro findings are promising, in vivo studies are needed to confirm the efficacy and safety of GC and WFA in a more complex biological environment. Animal models of pancreatic cancer could provide important information on the pharmacokinetics, toxicity, and therapeutic potential of this combination therapy.

Finally, the study highlights the importance of considering the heterogeneity of pancreatic cancer in developing therapeutic strategies. The differences in response between the PANC-1 and Hs766t cell lines underscore the need for personalized approaches to treatment, taking into account the specific characteristics of the cancer cells. Future research should focus on identifying biomarkers that can predict response to GC and WFA, allowing for more tailored and effective treatment strategies.

While gemcitabine has long been a mainstay in the systemic treatment of pancreatic cancer, its role is increasingly being overtaken by more effective combination regimens, such as FOLFIRINOX [6,7]. FOLFIRINOX, which combines fluorouracil, leucovorin, irinotecan, and oxaliplatin, has demonstrated greater efficacy compared to gemcitabine alone, particularly in advanced stages of pancreatic cancer [71]. However, despite these improved outcomes, resistance to FOLFIRINOX remains a significant challenge [72].

This study’s findings, which highlight the synergistic effects of GC and WFA, indicate that WFA could potentially be used alongside FOLFIRINOX components to overcome resistance. WFA’s ability to modulate key pathways, such as reactive oxygen species (ROS) production and NF-κB activity, may play a crucial role in sensitizing pancreatic cancer cells to FOLFIRINOX, particularly in cases where resistance has developed. WFA’s disruption of NF-κB signaling, a pathway frequently associated with chemoresistance, underscores its potential to enhance the efficacy of FOLFIRINOX by promoting apoptosis and reducing survival signals.

As the treatment landscape for pancreatic cancer continues to evolve, further research should explore the potential of combining WFA with FOLFIRINOX, with a focus on its ability to counteract resistance mechanisms. By targeting the ROS-NF-κB axis, WFA may offer a novel strategy to enhance the effectiveness of FOLFIRINOX, potentially leading to better outcomes for patients whose cancers have not responded to conventional therapies.

## 5. Conclusions

In conclusion, this study provides compelling evidence that the combination of gemcitabine and Withaferin A has significant potential as a treatment for pancreatic cancer. The synergy between these two agents in inhibiting proliferation, inducing apoptosis, reducing migration, altering cell stiffness, and increasing ROS levels suggests that they could be particularly effective when used together. These findings support the further exploration of GC and WFA as a combination therapy, both in pre-clinical studies and clinical trials, with the goal of improving outcomes for patients with pancreatic cancer.

## Figures and Tables

**Table 1:** Values of IC 10 and IC50. Data are expressed as a mean ± standard deviation.

Cell Line	GC		WFA	
	IC10	IC50	IC10	IC50
PANC-1	3.48 ± 0.36 [nM]	28.6 ± 2.16 [nM]	2.48 ± 0.24 [µM]	17.78 ± 1.23 [µM]
Hs766t	7.51 ± 0.68 [nM]	63.1 ± 3.74 [nM]	1.60 ± 0.18 [µM]	12.59 ± 0.98 [µM]

For the PANC-1 cell line, WFA demonstrated an IC50 of 17.78 µM (±1.23 µM), indicating a substantial reduction in cell proliferation, while GC showed a comparable inhibitory effect with an IC50 of 28.6 nM (±2.16 nM). In the case of the Hs766t cell line, WFA was particularly effective, achieving an IC50 of 12.59 µM (±0.98 µM), which suggests a strong suppression of proliferative activity. Conversely, GC exhibited a lesser impact on Hs766t cell proliferation compared to PANC-1 cells, with an IC50 of 63.10 nM (±3.74 nM), indicating a more varied response among the cell population. These findings illustrate the distinct influences of WFA and GC on the proliferative capacities of these two pancreatic cancer cell lines, providing valuable information for potential therapeutic interventions.

## Data Availability

The data presented in this study are available on request from the corresponding author.
